# Leaves of *Cedrela sinensis* Attenuate Chronic Unpredictable Mild Stress-Induced Depression-like Behavior via Regulation of Hormonal and Inflammatory Imbalance

**DOI:** 10.3390/antiox11122448

**Published:** 2022-12-12

**Authors:** Hye Rin Jeong, Jong Min Kim, Uk Lee, Jin Yong Kang, Seon Kyeong Park, Hyo Lim Lee, Jong Hyun Moon, Min Ji Kim, Min Ji Go, Ho Jin Heo

**Affiliations:** 1 Division of Applied Life Science (BK21), Institute of Agriculture and Life Science, Gyeongsang National University, Jinju 52828, Republic of Korea; 2 Division of Special Forest Products, National Institute of Forest Science, Suwon 16631, Republic of Korea; 3 Fermentation Regulation Research Group, World Institute of Kimchi, Gwangju 61755, Republic of Korea; 4 Korea Food Research Institute, Jeonju 55365, Republic of Korea

**Keywords:** *Cedrela sinensis*, chronic unpredictable mild stress, depression, mitochondrial function, inflammation, hormone changes

## Abstract

This study aimed to evaluate the protective effects of ethyl acetate fraction from *Cedrela sinensis* (EFCS) against chronic unpredictable mild stress (CUMS)-induced behavioral dysfunction and stress response in C57BL/6 mice. The physiological compounds of EFCS were identified as rutin, isoquercitrin, ethyl gallate, quercitrin, kaempferol-3-O-rhamnoside, and ethyl digallate, using UPLC-Q-TOF/MS^E^. To evaluate the neuroprotective effect of EFCS, H_2_O_2_^−^ and corticosterone-induced neuronal cell viability was conducted in human neuroblastoma MC-IXC cells. It was found that EFCS alleviated depression-like behavior by conducting the sucrose preference test (SPT), forced swimming test (FST), open field test (OFT), and tail suspension test (TST). EFCS inhibited mitochondrial dysfunction related to neuronal energy metabolism by regulating reactive oxygen species (ROS) levels, mitochondrial membrane potential (MMP), and ATP contents in brain tissue. In addition, the administration of EFCS regulated the stress hormones in serum. EFCS regulated stress-related indicators such as CRF, ACTH, CYP11B1, and BDNF. Moreover, EFCS downregulated the inflammatory responses and apoptosis proteins such as caspase-1, TNF-α, IL-1β, p-JNK, BAX, and p-tau in brain tissues. These results suggest that EFCS might be a potential natural plant material that alleviates CUMS-induced behavior disorder by regulating inflammation in brain tissue against CUMS-induced depression.

## 1. Introduction

Stress, a vocabulary word for expressing various anxious emotions, is an inevitable phenomenon that adversely affects daily life [1]. When the human body is continuously exposed to various stress, it is affected by disorders such as anxiety, depression, anxiety disorders, and immune-system-related disorders [2]. According to the World Health Organization (WHO), depressive disorders derived from chronic stress are one of the major causes of global disease, with representative characteristics of low self-esteem, fatigue, sleep disorders, and loss of control [3,4]. In general, the stress that can be tolerated in the body is properly regulated through the hypothalamic–pituitary–adrenal (HPA) axis, but excessive stress causes the abnormality of the axis [4]. It is reported that hypothalamic–pituitary–adrenal (HPA) axial hyperactivity is a major mechanism for abnormal pathways that occur in the body from stress [2,5]. Increased stress from various external stimuli activates corticotropin-releasing factor (CRF) receptors in the hypothalamus, causing the secretion of adrenocorticotropic hormone (ACTH), which releases glucocorticoids [6]. Abnormally increased glucocorticoid from chronic stress leads to HPA axis dysregulation and stimulates the imbalance of hormones, such as the suppression of dopamine, serotonin, and melatonin levels and the increase in cortisol and corticosterone, causing depressive behavior [7]. Increased stress hormone exposure affects neuroinflammation, neuronal loss, and dendritic atrophy in brain tissues and releases inflammatory cytokines to alter the neurotransmitter function of neurons by stimulating microglia [8,9,10]. Moreover, chronic stress causes oxidative stress and proinflammatory state by increasing inflammatory cytokines such as interleukin-1β (IL-1β) and tumor necrosis factor alpha (TNF-α) [11]. Inflammatory cytokines continuously produced from chronic stress interact with astrocytes involved in the structure and function of synapses and deteriorate the structure of neural circuits [12]. In addition, continuous exposure to stress is related to various diseases, such as infectious, autoimmune, and neurodegenerative diseases, which cause pathological problems and pain through complex interactions with inflammation, oxidative stress, and apoptosis [13]. In conclusion, exposure to chronic stress causes an imbalance in the secretion of various cytokines and the HPA axis and disturbs the homeostasis of inflammatory cytokines through vagus fiber activation, leading to various diseases such as depression [14,15].

*Cedrela sinensis* (*Toona sinensis*) is cultivated in East Asian countries such as Republic of Korea, China, and Japan [16]. Young leaves of *Cedrela sinensis* contain carotenes, vitamin B, and vitamin C [17]. In addition, *Cedrela sinensis* is rich in flavonoids, alkaloids, terpenes, and anthraquinones [18]. Recent studies on *Cedrela sinensis* have reported that it has antibacterial activity, skin moisturizing effects, and anti-inflammatory and analgesic effects [19,20,21]. The aqueous extracts of *Cedrela sinensis* leaves possess multiple activities such as antioxidation and antiproliferation [22]; however, there are few studies on *Cedrela sinensis* related to the protective effect against chronic-stress-induced depression.

The chronic unpredictable mild stress (CUMS) model consists of mild stressors that mimic the stresses commonly found in human life, and exposure to CUMS induces nerve damage, abnormal endocrine system activity, behavioral abnormalities, and depression [7]. The CUMS protocol is one of the animal models used to study the underlying mechanisms of depression and stress-induced behavior impairment and represents the structural and functional impairment of cerebral tissue in rodents [23]. In various CUMS models, it has been reported that chronic stress causes spatial learning deficit, CA3 dendritic atrophy, and decreased brain-derived neurotrophic factor (BDNF) expression through synaptic morphology changes [24,25,26]. Therefore, this study was conducted to evaluate the ameliorating effect of *Cedrela sinensis*, which presented considerable antioxidant capacities in a previous study (Table S1) in CUMS-induced C57BL/6 mice.

## 2. Materials and Methods

### 2.1. Chemicals

Folin–Ciocalteu reagent, Na_2_CO_3_, gallic acid, diethylene glycol, NaOH, rutin, 2,2′-azino-bis (3-ethylbenzothiazoline-6-sulfonic acid) (ABTS), NaCl, 2,4,6-tri(2-pyridyl)-1,3,5-triazine (TPTZ), HCl, FeCl_3_, Tris-HCl, FeSO_4_, ascorbic acid, trichloroacetic acid, thiobarbituric acid, Minimum Essential Medium (MEM), fetal bovine serum (FBS), penicillin, streptomycin, 3-(4,5-Dimethylthiazol-2-yl (MTT), sucrose, malate, pyruvate, mannitol, HEPES sodium salt, egtazic acid (EGTA), trichloroacetic acid (TCA) digitonin, 5,5,6,6-tetrachloro-1,1,3,3-tetraethylbenzimidazolylcarbocyanine iodide (JC-1), and solvents were obtained from Sigma-Aldrich Chemical Corp. (St. Louis, MO, USA). An ATP assay kit was purchased from Promega Corp. (Madison, WI, USA).

### 2.2. Sample Preparation

*Cedrela sinensis* used in this experiment was purchased from Seosan-si (Republic of Korea) in May 2020 and verified by the National Institute of Forest Science (Suwon, Republic of Korea). The sample was dried using a vacuum tray dryer (FDU-8612, Operon, Gimpo, Republic of Korea) and stored at –20 °C. Dried sample was extracted with 40% ethanol. The extracted sample was filtered with a filter paper (Advantec No. 2 330 mm, Advantec Co. Ltd., Tokyo, Japan), and concentrated using a vacuum rotary evaporator (N-N series, Eyela Co., Tokyo, Japan). The concentrated extract was sequentially fractionated with n-hexane, chloroform, and ethyl acetate. Ethyl acetate fraction from *Cedrela sinensis* (EFCS) was dried and stored frozen at −20 °C before use in each experiment.

### 2.3. Physiological Compound Analysis

To identify the physiologically bioactive compounds, the EFCS dissolved in 50% methanol was analyzed using ultra performance liquid chromatography quadrupole-time-of-flight mass spectrometry (UPLC-Q-TOF/MS^E^, Xevo G2-S, Waters Corp., Milford, MA, USA) with a BEH C_18_ column (100 × 2.1 mm, 1.7 μm; Waters Corp.). The mobile phases were solvent A (0.1% formic acid in distilled water) and solvent B (0.1% formic acid in acetonitrile) during analysis, and gradient conditions were as follows: 0% B at 0–0.5 min, 0–100% B at 0.5–8 min, 100% B at 8–8.5 min, 100–0% B at 8.5–10 min, and 0% B at 10–12 min. The conditions used for the electrospray ionization (ESI) source were as follows: ramp collision energy, 20–45 V; oven temperature, 40 °C; capillary voltage, 3 kV; pressure of nebulizer, 40 psi; fragmentor, 175 V; cone voltage, 40 V; mass range, 50–1500 *m*/*z*. The UPLC-Q-TOF/MS^E^ system was analyzed using data analysis software (Waters MassLynx™. Waters Corp.).

### 2.4. Evaluation of Neuronal Protective Effect

#### 2.4.1. Cell Culture and Treatment

MC-IXC cells (ATCC^®®^-CRL-2270^TM^) with the characteristics of human neuroblastoma cell lines were acquired from the American Type Culture Collection (ATCC, Manassas, VA, USA) and incubated in MEM medium with 10% FBS, 50 units/mL penicillin, and 100 μg/mL streptomycin in the conditions of 5% CO_2_ at 37 °C.

#### 2.4.2. Cell Viability

The cell viability was estimated by the MTT assay [27]. MC-IXC cells (10^4^ cells/well) were treated with the EFCS. After 24 h or 3 h, the cells were treated with H_2_O_2_ or corticosterone and incubated for 24 h or 3 h. Finally, the cells were reacted with 5 mg/mL of MTT solution for 3 h. The contents of the MTT formazan were measured using a microplate reader (Epoch 2, BioTek Instruments, Inc., Winooski, VT, USA) at a test wavelength of 570 nm and reference wavelength of 690 nm.

### 2.5. Chronic Unpredictable Mild Stress (CUMS) Procedures

**Figure 1:**
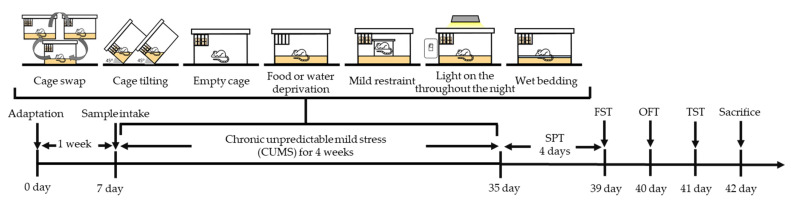
Schematic experimental design in the chronic unpredictable mild stress (CUMS)-induced mice. SPT, sucrose preference test; FST, forced swim test; OFT, open field test; TST, tail suspension test.

The CUMS protocol was adapted from the procedure described in previous research [28]. The mice were randomly exposed to 7 stresses, one per day for a week, and the total period was 4 weeks. The order of CUMS procedures was conducted according to Excel function (=ramdbetween) (Microsoft Office 2016, Microsoft Corp., Redmond, WA, USA). The CUMS methods and the experimental design are presented in Table 1 and Figure 1, respectively.

### 2.6. Animal Design

The experimental C57BL/6 mice (male, 4 weeks) were purchased from Samtako (Osan, Republic of Korea). All procedures of the animal housing and experiment followed the guidelines of the Animal Care and Use Committee of Gyeongsang National University (certificate: GNU-200803-M0048, approved on 3 August 2021) and were performed in accordance with the Policy of the Ethical Committee of Ministry of Health and Welfare, Republic of Korea. These were accommodated in standard laboratory conditions (22 ± 2 °C, 55% humidity, 12 h light/dark cycle) where fodder and water were freely available. The mice were divided into normal control (NC) group (non-CUMS treatment, vehicle administration), CUMS (CUMS treatment, vehicle administration) group, EFCS 20 group (CUMS treatment, EFCS (20 mg/kg of body weight) administration), and EFCS 50 group (CUMS treatment, EFCS (50 mg/kg of body weight) administration) (*n* = 10/group; 5 for in vivo behavioral test and 5 for mitochondrial test). After adaptation for 1 week to the environment, experimental study was conducted. The mice were orally fed a sample dissolved in drinking water through a stomach tube once a day for 4 weeks.

### 2.7. Behavioral Tests

#### 2.7.1. Sucrose Preference Test (SPT)

To measure the SPT, on the first day, mice were individually accommodated in the cage during adaptation, and two bottles containing 1% sucrose solution were placed in the cage for 24 h. On the second day, one of the sucrose bottles was changed to drinking water with nonsucrose solution for 24 h. On the third day, to prevent their preference for location, the positions of the two bottles (left/right) were changed and food and water were removed. Lastly, the consumption of each bottle was measured on the fourth day. The sucrose preference was calculated using the following formula [29].

$$\mathrm{Sucrose}\;\mathrm{preference}=\frac{\mathrm{sucrose}\;\mathrm{consumption}}{\mathrm{sucrose}\;\mathrm{consumption}+\mathrm{water}\;\mathrm{consumption}}\;\text{×}\;100\%$$

#### 2.7.2. Forced Swimming Test (FST)

The open cylinder container (50 cm height × 20 cm diameter) was composed of acrylic glass material, and the water depth was maintained at a level of 15 cm at 25 ± 1 °C. Each mouse was determined to have immobility and activity time, and the movement of the mice was recorded for 6 min using a video tracking system (Smart 3.0, Panlab, Barcelona, Spain) [30].

#### 2.7.3. Tail Suspension Test (TST)

A white acrylic box (50 cm width × 50 cm length × 50 cm height) with a long iron rod placed on top of the acrylic box was used in the experiment. Each mouse’s tail was placed in the center of the long iron rod, and their movement was recorded for 5 min using a video tracking system (Smart 3.0, Panlab) [31].

#### 2.7.4. Open Field Test (OFT)

To measure the OFT, the OFT was conducted in a white acrylic box (50 cm width × 50 cm height × 50 cm length). Before the experiment, the mice were located in the designated peripheral area, and the activity of each mouse in the central (within 25 cm width × 25 cm length) area and peripheral areas was recorded for 5 min using a video tracking system (Smart 3.0, Panlab) [32].

### 2.8. Preparation of Brain Tissues

After in vivo tests, whole brain and hypothalamus tissues were collected from the sacrificed mice. The whole brain tissues were used for mitochondrial evaluation and Western blot tests, and hypothalamus tissues were used for hormonal tests.

### 2.9. Mitochondrial Activity

#### 2.9.1. Mitochondrial Isolation

Brain tissue was homogenized using a mitochondria isolation (MI) buffer (215 mM mannitol, 75 mM sucrose, 0.1% bovine serum albumin (BSA), 20 mM HEPES sodium salt, and 1 mM EGTA, pH 7.2) and centrifuged at 1300× *g* for 5 min. The supernatant was centrifuged again at 13,000× *g* for 10 min to obtain pellets. An isolation buffer containing 1 mM EGTA and 0.1% digitonin was added to the obtained pellets and left in ice for 5 min, which was centrifuged at 13,000× *g* for 15 min at 4 °C. The obtained pellets were mixed with isolation buffer and centrifuged at 10,000× *g* for 10 min, and mitochondrial activity was evaluated using the finally obtained pellets.

#### 2.9.2. Measurement of Mitochondrial Reactive Oxygen Species (ROS) Contents

To assess the amount of ROS in mitochondria, KCl-based respiration buffer (125 mM potassium chloride, 2 mM potassium phosphate monobasic, 2.5 mM malate, 20 mM HEPES, 1 mM magnesium chloride, 5 mM pyruvate, and 500 μM EGTA, pH 7.0) was mixed with DCF-DA for 20 min. This reaction solution was measured at excitation wave 485 nm and emission wave 535 nm using a microplate reader (Epoch2, BioTek, Winooski, VT, USA) [33].

#### 2.9.3. Measurement of the Mitochondrial Membrane Potential (MMP)

To investigate the MMP, the mitochondrial extract was mixed with MI buffer containing 5 mM pyruvate and 5 mM malate, and then 1 μM JC-1 was reacted in the dark for 20 min. The reaction solution was measured at excitation wave 530 nm and emission wave 590 nm using a fluorescence photometer (Infinite F200, Tecan, Männedorf, Switzerland) [33].

#### 2.9.4. Measurement of Mitochondrial ATP Contents

To measure the ATP content in the mitochondrial extract extracted from brain tissue, the extract was centrifuged at 13,000× *g* for 10 min, and the pellet was mixed with 1% TCA and reacted on ice for 10 min. After mixing with 25 mM tris-acetate buffer (pH 7.7) at 10,000× *g* for 15 min, the supernatant was used to measure the ATP content, which was performed using an ATP assay kit (Promega Corp., Madison, WI, USA) using a luminometer (GloMax-Multi Detection System, Promega Corp., Madison, WI, USA).

### 2.10. Hormonal Analysis

Hormone metabolites of hypothalamus were analyzed with an ultraperformance liquid chromatography ion mobility separation quadrupole time-of-flight/tandem mass spectrometry (UPLC-Q-TOF/MS^2^, Waters Corp., Milford, MA, USA) with positive ESI mode. The samples were injected into an Acquity UPLC BEH C_18_ column (100 × 2.1 mm, 1.7 μm; Waters Corp., Milford) equilibrated with water containing 0.1% formic acid and eluted in a gradient with acetonitrile containing 0.1% formic acid at a flow rate of 0.5 mL/min for 30 min. The voltages of the capillary were 2.78 kV, and MS spectra of the metabolites were collected in the 50–1000 *m*/*z* range using a collision energy ramp from 10–30 eV. All MS data obtained were analyzed using MassLynx software (Waters Corp.)

### 2.11. Western Blot Analysis

The expression of proteins was measured in brain tissue. Proteins were extracted from the extracted tissue using a cell/tissue lysis buffer (ProtinEx™ Animal cell/tissue, Gene All Biotechnology Co., Ltd., Seoul, Republic of Korea) containing 1% protease inhibitor. The protein extract was centrifuged at 13,000× *g* for 10 min to obtain a supernatant, and the protein was quantified relative to the supernatant using Bradford method [34]. The protein extract was separated by sodium dodecyl sulfate polyacrylamide gel electrophoresis (SDS-PAGE) and transferred to a poly-vinylidene difluoride (PVDF) membrane. Thereafter, the membrane was blocked using 5% skim milk for 1 h, and the primary antibody was reacted overnight. After that, a secondary antibody was reacted at room temperature for 1 h, the membrane was reacted with ECL (ProNA™ECL Ottimo, TransLab, Daejeon, Republic of Korea) solution, and the luminescence density was detected using ChemiDoc (iBrightTM CL1000 instrument, Invitrogen, Carlsbad, CA, USA). The detected band density was quantified using ImageJ software (National Institutes of Health, Bethesda, MD, USA), and the density value of each factor was divided by the density value of β-actin. The antibody information is presented in (Supplemental Data Table S2).

### 2.12. Statistical Analysis

All experiment results were repeated and expressed as mean ± standard deviation, and each mean value was analyzed for variance using SAS software (v9.4, SAS Institute, Cary, NC, USA) and Duncan’s multiple range test to verify the significance between samples within 5% level. All experimental data were evaluated for normality and variance homogeneity with the Shapiro–Wilk and Levene’s variance homogeneity tests. The statistical significance of the data is indicated with different lowercase letters, and means with the same letter are not significantly different.

## 3. Results

### 3.1. Physiological Compound Using UPLC Q-TOF/MS^E^

**Figure 2:**
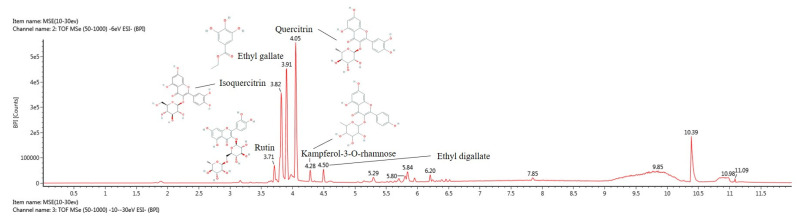
UPLC Q–TOF/MSE chromatography in negative ion mode of ethyl acetate fraction from *Cedrela sinensis* (EFCS).

The bioactive compounds of EFCS were qualitatively identified using UPLC-Q-TOF/MS^E^ analysis (Figure 2 and Table 2). The MS^E^ spectra were continuously obtained in negative ion mode (M−H)^−^ as compound 1: 609.14 *m*/*z* (retention time (RT: 3.71 min); compound 2: 463.09 *m*/*z* (RT: 3.82 min); compound 3: 197.04 *m*/*z* (RT: 3.91min); compound 4: 447.09 *m*/*z* (RT: 4.05 min) compound 5: 431.10 *m*/*z* (RT: 4.28 min); and compound 6: 349.05 *m*/*z* (RT: 4.50 min). These compounds were tentatively identified as rutin (compound 1), isoquercitrin (compound 2), ethyl gallate (compound 3), quercitrin (compound 4), kaempferol-3-O-rhamnoside (compound 5), and ethyl digallate (compound 6), according to a library software program (Waters MassLynx™, Waters Corp.) and previous studies [35,36,37,38,39,40].

### 3.2. Neuroprotective Effect

**Figure 3:**
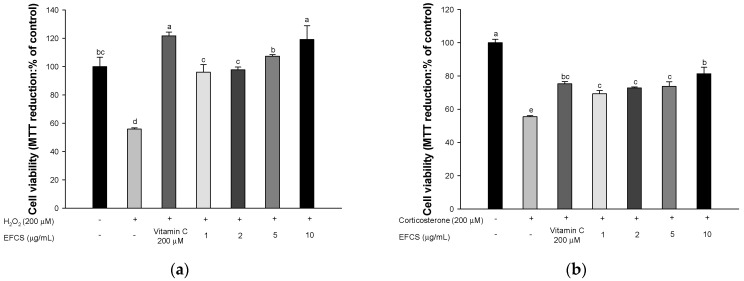
Neuroprotective effects of ethyl acetate fraction from *Cedrela sinensis* (EFCS) in MC−IXC cells. H_2_O_2_^−^ induced cell viability (**a**) and corticosterone-induced cell viability (**b**). Results shown are mean ± SD (*n* = 5). Data are statistically represented at *p* < 0.05, and different lowercase letters indicate statistical significance. Means with the same letter are not significantly different.

To evaluate the neuroprotective effect of EFCS, cell viability was measured using MTT analysis in human neuroblastoma MC-IXC cells (Figure 3). The sample concentration was determined through a prior study to determine the concentration that does not affect the MC-IXC cells (Figure S1). The cell viability of the H_2_O_2_^−^ and corticosterone-induced groups (55.85% and 55.47%, respectively) was reduced compared to the normal control group (100%); however, the vitamin C- and EFCS-treated groups presented increased cell viability (vitamin C, 121.74% and 75.36%; 10 μg/mL, 119.12% and 81.38%; 5 μg/mL, 107.31% and 73.70%, respectively) compared to that of the H_2_O_2_^−^ and corticosterone-induced groups.

### 3.3. Behavioral Test

**Figure 4:**
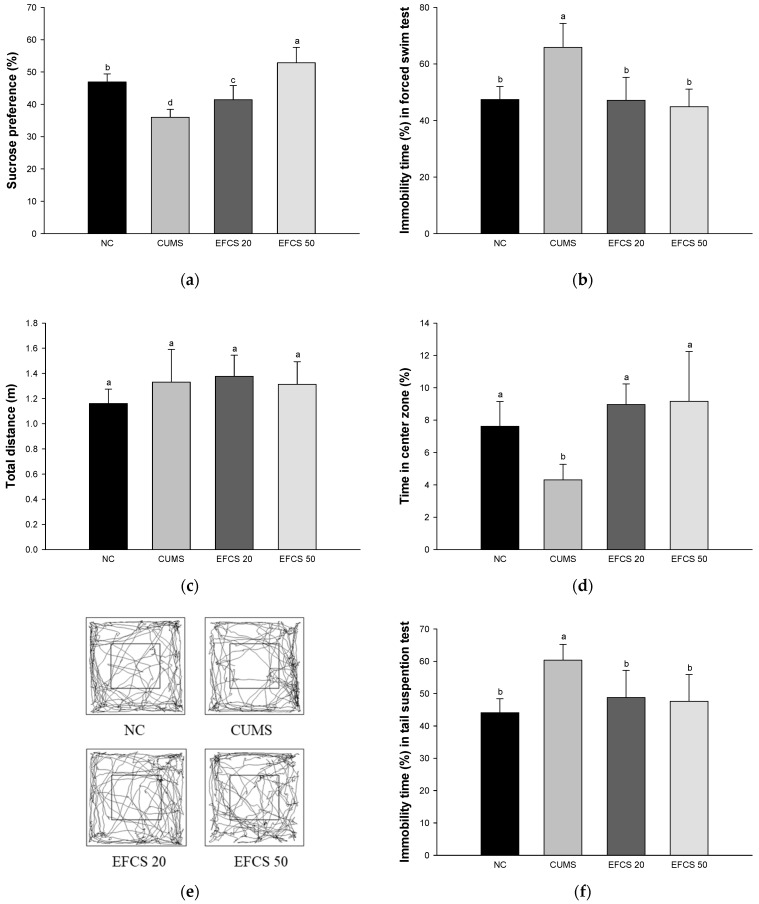
Effects of ethyl acetate fraction from *Cedrela sinensis* (EFCS) on depression-related behaviors in mice with CUMS exposure. Percentage of sucrose preference in sucrose preference test (SPT) (**a**), percentage of immobility time in forced swimming test (FST) (**b**), the total distance in open field test (OFT) (**c**), percentage of center zone in OFT (**d**), path tracing on the probe trial (**e**) in OFT and percentage of immobility time in tail suspension test (TST) (**f**). Results shown are mean ± SD (*n* = 5). Data are statistically represented at *p* < 0.05, and different lowercase letters indicate statistical significance. Means with the same letter are not significantly different.

In the results of SPT, the sucrose preference was significantly decreased in the CUMS group (36.01%) compared with the NC group (46.91%), whereas the EFCS groups presented increased sucrose preference compared to that of the CUMS group (EFCS 20, 41.27%; EFCS 50, 52.85%, respectively) (Figure 4a).

In the results of FST, the immobility time of CUMS group (47.43%) was significantly increased compared to the NC group (65.86%). On the other hand, the EFCS groups (EFCS 20, 47.14%; EFCS 50, 44.86%, respectively) showed a significant decrease compared to that of the CUMS group (Figure 4b).

In the results of OFT, there was no significant difference in total distance between groups (Figure 4c). The time spent in the center zone of the CUMS group (4.31%) was decreased compared to the NC group (7.61%); however, the time spent in the center zone of the EFCS groups (EFCS 20, 8.96%; EFCS 50, 9.16%, respectively) was increased compared to that of the CUMS group (Figure 4d). In the results of the path tracing in OFT, the CUMS group tended to mainly stay at the outside of the field compared to the NC group, whereas the EFCS groups tended to mainly stay in the center zone compared to the CUMS group (Figure 4e).

In the results of TST, the immobility time of the CUMS group (44.08%) was significantly increased compared to the NC group (60.33%) (Figure 4f). On the other hand, the immobility time of the EFCS group (EFCS 20, 48.78%; EFCS 50, 47.62%, respectively) decreased compared to the immobility time of the CUMS group.

### 3.4. Mitochondrial Activity

**Figure 5:**
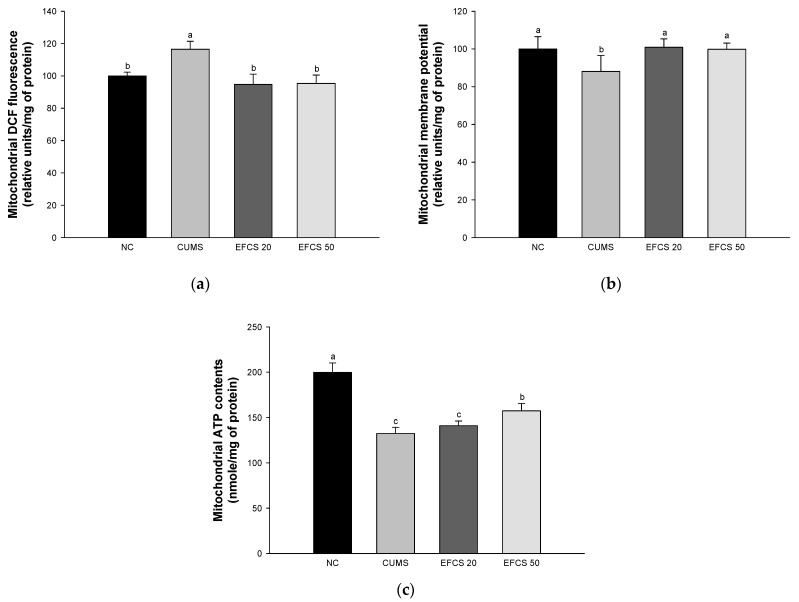
Effect of ethyl acetate fraction from *Cedrela sinensis* (EFCS) on CUMS-induced mitochondrial dysfunction. ROS production contents (**a**), mitochondrial membrane potential (MMP) contents (**b**), and mitochondrial ATP contents (**c**). Results shown are mean ± SD (*n* = 5). Data are statistically represented at *p* < 0.05, and different lowercase letters indicate statistical significance. Means with the same letter are not significantly different.

To assess the ameliorating effect of EFCS on CUMS-induced mitochondrial dysfunction, ROS production, MMP, and ATP levels were investigated (Figure 5). The CUMS group (116.50%) increased ROS production compared to the NC group (100%). On the other hand, the EFCS groups (EFCS 20, 94.68%; EFCS 50, 95.36) considerably decreased ROS reduction compared with the CUMS group. The MMP of the CUMS group (88.08%) decreased compared with the NC group (100%), while both EFCS groups (EFCS 20, 100.79%; EFCS 50, 98.41%) enhanced membrane potential compared to the CUMS group. The ATP levels of the CUMS group (132.50%) increased compared to the NC group (188.48%). For the EFCS 20 group (141.07%), there was no significant difference compared to the CUMS group; however, the EFCS 50 group (157.35%) confirmed a significant increase compared to the CUMS group.

### 3.5. Hypothalamic Hormone Change

**Figure 6:**
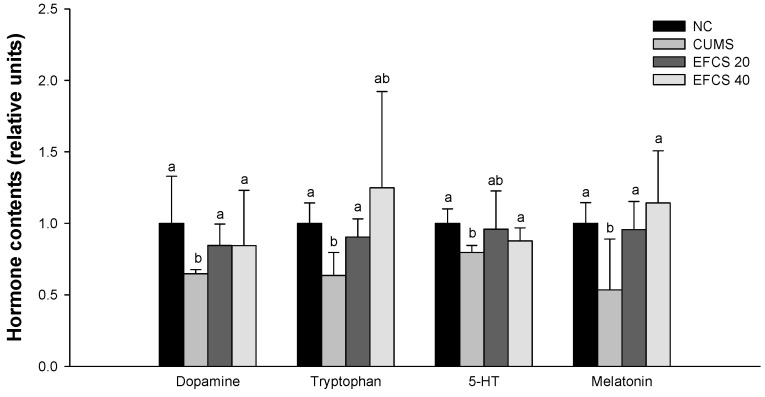
Effect of ethyl acetate fraction from *Cedrela sinensis* (EFCS) on dopamine, tryptophan, 5-HT, and melatonin contents in hypothalamus tissues. Results shown are mean ± SD (*n* = 3). Data are statistically represented at *p* < 0.05, and different lowercase letters indicate statistical significance. Means with the same letter are not significantly different.

The relative hormone contents in the hypothalamus tissues are shown in Figure 6. The dopamine, tryptophan, 5-hydroxytryptamine (serotonin, 5-HT), and melatonin levels of the CUMS group (64.85%, 63.54%, 79.72%, and 53.46%, respectively) were reduced compared to the NC group (100%); however, those levels of the EFCS 20 group (84.57%, 90.48%, 95.96%, and 95.63%, respectively) and EFCS 50 group (84.50%, 124.82%, 87.74%, and 114.20%, respectively) increased compared to the CUMS group.

### 3.6. Stress-Related Pathway

**Figure 7:**
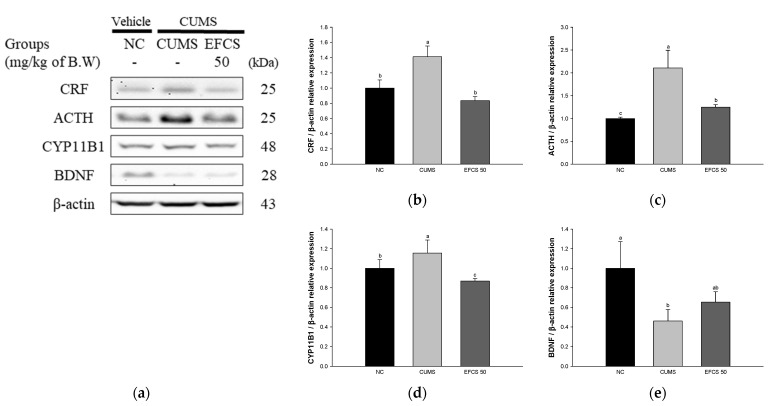
Effect of ethyl acetate fraction from *Cedrela sinensis* (EFCS) on CUMS-induced stress pathway in brain tissue. Western blot images (**a**), protein expression levels of CRF (**b**), ACTH (**c**), CYP11B1 (**d**), and BDNF (**e**) in brain tissues. Results shown are mean ± SD (*n* = 3). Data are statistically considered at *p* < 0.05, and different lowercase letters indicate statistical significance. Means with the same letter are not significantly different.

To demonstrate the antistress effect of EFCS in CUMS-induced mouse brain tissue, the protein expression of CRF, ACTH, cytochrome P450 Family 11 Subfamily B Member 1 (CYP11B1), and BDNF were measured by Western blot analysis (Figure 7). The protein expressions of CRF (141.17%), ACTH (210.84%), and CYP11B1 (115.58%) in the CUMS group were significantly upregulated compared to those in the NC group (100%); however, the EFCS 50 group statistically downregulated CRF (83.58%), ACTH (125.04%), and CYP11B1 (88.55%) compared to the CUMS group. The BDNF (46.04%) in the CUMS group was significantly downregulated compared to the NC group (100%); however, the EFCS 50 group statistically upregulated BDNF (65.35%) compared to the CUMS group.

### 3.7. Protein Expression of Neuroinflammation

**Figure 8:**
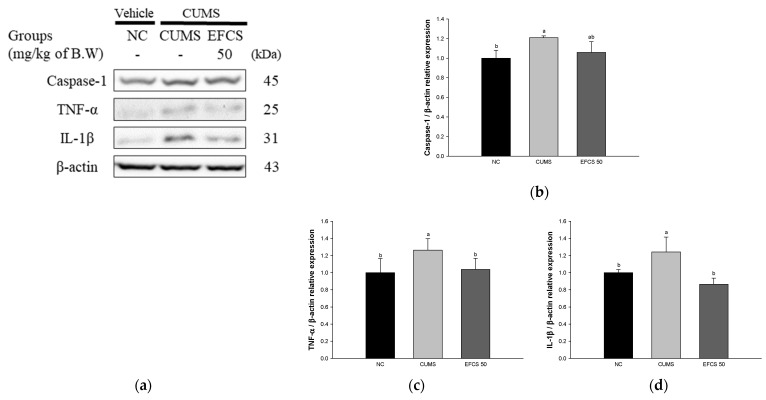
Effect of ethyl acetate fraction from *Cedrela sinensis* (EFCS) on CUMS-induced inflammation pathway in brain tissue. Western blot images (**a**), protein expression levels of caspase-1 (**b**), TNF-α (**c**), and IL-1β (**d**) in brain tissues. Results shown are mean ± SD (*n* = 3). Data are statistically considered at *p* < 0.05, and different lowercase letters indicate statistical significance. Means with the same letter are not significantly different.

To confirm the effect of EFCS on CUMS-induced inflammation in brain tissue, the protein expression of caspase-1, TNF-α, and IL-1β was measured by Western blot analysis (Figure 8). The protein expression of caspase-1 (120.83%), TNF-α (126.28%), and IL-1β (124.16%) of the CUMS group was significantly upregulated compared with the NC group (100%). On the other hand, the EFCS groups presented decreased protein expression of caspase-1 (105.94%), TNF-α (103.80%), and IL-1β (86.38%) compared to the CUMS group.

### 3.8. Protein Expression of Apoptosis

**Figure 9:**
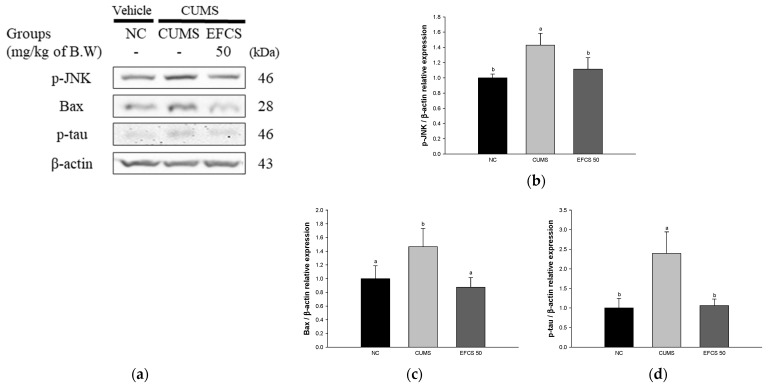
Effect of ethyl acetate fraction from *Cedrela sinensis* (EFCS) on CUMS-induced apoptosis pathway. Western blot images (**a**), protein expression levels of p-JNK (**b**), Bax (**c**), and p-tau (**d**) in brain tissues. Results shown are mean ± SD (*n* = 3). Data are statistically considered at *p* < 0.05, and different lowercase letters indicate statistical significance. Means with the same letter are not significantly different.

To confirm the protective effect of EFCS in CUMS-induced apoptosis in brain tissue, the protein expression of B-cell lymphoma 2 (BCl-2)-associated X protein (Bax), phosphorylated c-Jun N-terminal kinases (JNK), and phosphorylated tau (p-tau) were measured by Western blot analysis (Figure 9). The protein expression of Bax (146.61%), p-JNK (142.99%), and p-tau (239.54%) of the CUMS group was considerably upregulated compared to the NC group (100%); however, the protein expression of Bax (87.46%), p-JNK (111.39%), and p-tau (106.03%) of the EFCS groups was downregulated more than the CUMS group.

## 4. Discussion

The chronic stress response develops when homeostasis is perceived as being threatened by internal or external stimulation, and it is involved in the damage of several brain structures, including the hypothalamus, prefrontal cortex, and hippocampus [41]. Inflammatory cytokines stimulated by stress hormones play a central role in the immune system and inflammatory reactions, and these cytokines can cause serious changes in behavior, including the onset of depressive symptoms such as sad mood, discomfort, and fatigue [10]. The specific mechanisms involved in the ameliorating effects of chronic-stress-induced inflammatory response of *Cedrela sinensis* are not clear. Therefore, this study was performed to confirm the antidepression effect by measuring the inflammatory response induced by CUMS and the stress factors interacting pathophysiologically with the inflammatory response.

When the body is stimulated by external stress, a large amount of stress hormones, such as cortisol and corticosterone, is secreted into the body due to the excitation of the HPA axis [42]. A high concentration of corticosterone binds to glucocorticoid receptors (GR) in hippocampal tissue and stimulates Ca^2+^ channels and glutamate N-methyl-D-aspartate (NMDA) receptors [43]. It has been reported that continuous stimulation of stress hormones in neurons induces production of oxidative stress, alteration of the dendritic trees of hippocampal neurons, excitotoxicity, and ultimately cell death [44]. Similar to these results, the treatment of H_2_O_2_ as the oxidative stress and corticosterone led to cell viability in human neuroblastoma MC-IXC cells, but EFCS treatment protected cellular cytotoxicity induced by H_2_O_2_ and corticosterone (Figure 3). In a previous study, limonoids contained in fresh young leaves and buds of *Toona sinensis* protected against hydroxydopamine-induced cellular cytotoxicity in SH-SY5Y cells [45]. Leaves from *Cedrela sinensis* inhibited neurotoxicity by reducing cytosolic Ca^2+^ levels, glutamate release, and intracellular ROS production in primary cortical neuron cultures [46]. In addition, ethyl gallate, one of the bioactive compounds in EFCS, significantly suppressed H_2_O_2_^−^ induced cytotoxicity by regulating nuclear factor erythroid-2-related factor 2 (Nrf2) and the apoptosis signaling pathway in PC12 cells [47]. EFCS contains various flavonoids structurally based on quercetin and kaempferol (Table 2 and Figure 2). These compounds with the position of hydroxy group(s) on the A-ring of flavonoid can significantly suppress stress-induced cytotoxicity more than the structure without the hydroxy group(s). In particular, flavone C-glucosides, such as rutin, quercitrin, and isoquercitrin, presented considerable antidepressant effects [48]. In general, ingested quercetin derivates are converted to quercetin through the breakdown of sugar in the intestine, and quercetin is absorbed in the form of sulfate or glucuronate conjugates [49]. These metabolites easily pass through the BBB and can effectively protect brain neurons from various stresses [50]. Therefore, EFCS showed protective effects against oxidative-stress- and corticosterone-induced neuronal cytotoxicity from the various flavonoid compounds.

The activation of the HPA axis is the most well-known mechanism related to stress and signal pathways in brain tissue [7]. In particular, CRF is released from neuronal cells located in the paraventricular nucleus by chronic stress stimulation, and the released CRF is secreted into a hypophyseal portal system to promote the secretion of ACTH in the anterior pituitary gland [51]. ACTH reaches the adrenal cortex through the bloodstream and secretes glucocorticoids. The secreted glucocorticoids act primarily at the GR, preventing the further release of ACTH through negative feedback from the pituitary gland and the further release of glucocorticoids from the adrenal cortex [52]. The initial response to stress is for survival, but in the case of chronic stress exposed for a long period of time, HPA axis abnormality occurs due to an increase in cortisol level that is not properly regulated in response to negative feedback [53]. The increase in cortisol level causes atrophy of dendrites in the hippocampus, reduction of hippocampus volume, reaction of inhibiting neurogenesis in gyrus granule neurons, dendritic reduction of the medial prefrontal cortex, and the stimulation of inflammatory cytokines secretion [54]. In addition, inflammatory cytokines stimulated by chronic stress easily reach the brain through the blood–brain barrier (BBB) and damage sensory microneurons, which promotes similar behaviors such as depression [14]. In addition, inflammatory cytokines activate CRF of the paraventricular nucleus and alter cortisol upregulation and BDNF, thereby destroying the plasticity of the synapse [48]. In addition, inflammatory cytokines reduce the synthesis of monoamine neurotransmitters through indoleamine 2,3-dioxygenase and the mitogen-activated protein kinase (MAPK) pathway [55]. It has been reported that these inflammatory hormone abnormalities increase the expression of depression or depression-like behavior [11]. In this study, we confirmed that the CUMS group showed depression-like behavior by measuring the SPT, FST, and TST, but the administration of EFCS suppressed the behavioral abnormalities (Figure 4). Rutin, a bioactive compound of EFCS, reduced depression-like behavior in the OFT, FST, and SPT experiments [56]. Treatment with quercetin reduced behavioral dysfunction and abnormality by reducing the immobility time in FST and TST and chronically restrained depression [57]. Quercitrin induced an immediate and sustained antidepressant effect in LPS-treated mice [58]. In addition, kaempferol isolated from *Opuntia ficus-indica* var. *saboten* ameliorated chronic restraint stress by TST and FST by increasing the expression level of hypothalamic proopiomelanocortin, regulating the α-melanocyte-stimulating hormones (α-MSH), ACTH, and β-endorphin [48]. Therefore, it was confirmed that EFCS containing quercetin and kaempferol derivatives significantly improved behavioral dysfunction in CUMS-induced mice.

Mitochondria play an essential role in maintaining cell homeostasis, producing intracellular signaling, and regulating ROS [59]. When ROS is properly regulated, it plays an essential role in cell survival and signaling [60]; however, an imbalance of hormones and oxidative stress from chronic stress damages the antioxidants in neuronal cells, and leads to the excessive production of free radicals, mitochondrial dysfunction, and apoptosis pathways [61]. In particular, mitochondrial dysfunction is associated with the vulnerability of depression [62]. An increase in stress hormones by CUMS inhibited the respiration of mitochondria in the hippocampus, cortex, and hypothalamus, and reduced MMP [63]. In addition, mitochondria are easily oxidized by large amounts of metabolites generated by stress hormones, and they use enormous energy in controlling struggle and escape reactions [64]. In particular, the brain is vulnerable to stress because of its high GR that reacts to stress [65]. Long-term exposure to stress hormones such as glucocorticoid reacts with GR, and this complex increases the production of free radicals, respiratory dysfunction, ATP deficiency, and structural abnormalities in mitochondria [66]. In addition, this complex leads to lipid peroxidation and mitochondrial DNA damage, and initiates signals of apoptosis [67]. On the other hand, intake of EFCS protected against the production of ROS, and decreased MMP and ATP production in the brain mitochondria of CUMS-induced mice (Figure 5). According to a previous study, *Cedrela sinensis* leaf extract inhibited ROS generation and Ca^2+^ concentration in primary cultured cortical rat brain [46]. Rutin also inhibited ROS generation and increased MMP in amylin-indicated neuronal SH-SY5Y cells [66]. It has also been reported that rutin promotes mitochondrial biosynthesis by regulating various transcription factors such as proliferator-activated receptor gamma coactivator-1 alpha (PGC-1α), nuclear respiratory factor 1 (Nrf-1), and mitochondrial transcription factor A (TFAM) by regulating the silent information regulator, the two homolog 1 (SIRT1) signaling pathway [67]. Moreover, ethyl gallate protected against H_2_O_2_-induced mitochondrial damage by regulating caspase−9/−3 activation and MMP depletion [47]. Consequently, the administration of EFCS suppressed mitochondrial dysfunction by regulating ROS production, MMP, and ATP contents, and it can help maintain the mitochondrial function.

The most widely known hypothesis of major depressive disorder is the depletion of monoamines such as norepinephrine, dopamine, and 5-HT in the central neuronal system [68]. Chronic stress induces excessive secretion of serum cortisol and glucocorticoid [69]. It is reported that the increased glucocorticoid stimulated by chronic stress induces the abnormality of the HPA axis balance, which is known as an early sign of depression [14]. The imbalance of the HPA axis disrupts tryptophane absorption in intestinal tissue [53]. Tryptophan is involved in 5-HT and melatonin synthesis, and it can be easily absorbed into the brain with a high affinity for BBB transport [70]. The tryptophan is converted to 5-hydroxyptophan by the tryptophan hydroxylase enzyme, and finally, 5-hydroxyptophan is converted to 5-HT by the aromatic amino acid decarboxylase enzyme [70]. However, chronic stress decomposes 5-HT to 5-hydroxyindoleacetic acid (5-HIAA) through monoamine oxide and aldehyde hydrogenase, and decomposition of 5-HT continuously leads to HPA axis imbalance [71]. In addition, the decomposition of 5-HT induces the exhaustion of melatonin converted by 5-HT in the pineal gland and a decrease in melatonin disrupts the regulation of sleep rhythm [72]. In addition, chronic stress reduces the dopamine and norepinephrine levels and causes a reduction of arousal state, low energy, carelessness, and cognitive deficit [70,72]. Ultimately, a continuous increase in stress increases cortisol, causing a vicious cycle of tryptophan reduction, 5-HT reduction, and increased cortisol [73]. In this study, the exposure of chronic stress caused depletion of monoamine hormones; however, EFCS increased the levels of dopamine, tryptophan, 5-HT, and melatonin (Figure 6). It has been reported that *Cedrela sinensis* leaf is rich in hormonal precursor amino acids, such as tryptophan, tyrosine, and glutamic acid, that are associated with stress reduction [74,75]. These amino acids are absorbed into the body and converted to serotonin, melatonin, dopamine, and γ-aminobutyric acid (GABA), and they suppress stress and help to suppress the imbalance of the HPA axis [72,73]. Rutin, a physiological compound of EFCS, significantly increased levels of serum dopamine, 5-HT, and norepinephrine in a mild-hyperhomocysteinemia-induced rat model [76]. It has been reported that consumption of quercitrin is mediated by 5-HT receptors to modulate monoamine neurotransmitter levels, helping to treat anxiety disorders by increasing 5-HT and dopamine and reducing their metabolites such as 5-hydroxy-3-indoleacetic acid, 3,4-dihydroxyphenylacetic acid, and homovanillic acid [77]. Therefore, EFCS containing various amino acids and physiological phenolic compounds might improve stress levels by supplementing hormonal precursors and regulating stress-related hormone imbalance induced by CUMS.

The main route of stress response in the brain and peripheral immune system is the activation of nuclear factor kappa-light-chain-enhancer of activated B cells (NF-κB) via Toll-like receptors (TLRs), resulting in the production of inflammatory cytokines which can easily pass through the BBB [14]. Upregulated cytokines induce changes in neurological secretion function such as HPA axis activation, neutrophil activation, and apoptosis changes in the sympathetic nervous system [41]. They also affect the activation of CRF, which releases ACTH and cortisol in brain tissues [11]. CRF expressed in the cerebral cortex, hippocampus, and locus coeruleus in the brainstem is the main initiator of chronic-stress-induced HPA reactions [78]. CRF stimulates the production of ACTH in the anterior lobe of the pituitary gland and increases the secretion of ACTH reaching the adrenal cortex [79]. In the central neuronal system, ACTH increases the expression of CYP11B1, converting 11-deoxycorticosterone to corticosterone in the hypothalamus and cerebral cortex [80,81]. In addition, increased corticosterone activates the HPA axis to stimulate systemic cortisol levels, and the destruction of synaptic plasticity through the decrease in BDNF [14]. Similar to these results, CUMS treatment induced increased expression of CRF, ACTH, and CYP11B1 and decreased expression of GR and BDNF in the brain; however, EFCS significantly regulated the expression of CRF, ACTH, and CYP11B1 (Figure 7). Rutin, one of the flavonoids in EFCS, suppressed the hyperactivity of the HPA axis, decreasing the levels of ACTH and cortisol in the plasma of CUMS-induced rats [56]. In addition, quercetin ameliorated the depression-like behavior dysfunction via regulation of the cAMP response element-binding protein (CREB)/BDNF pathway and synaptic function in hippocampal tissues [58]. In addition, quercetin protected against hippocampal neurogenesis by regulating the Forkhead box transcription factor G1 (FoxG1)/CREB/BDNF pathway in CUMS-induced ICR mice [82]. Similarly, this result suggests that the protective effect of EFCS on behavioral disorders is represented by various quercetin and quercetin derivatives with a variety of physiological activities in EFCS. Thus, EFCS, which contains large amounts of various bioactive compounds, can be a potential functional ingredient for antidepressants by regulating stress-related factors.

The imbalance of hormones also increases inflammation and the production of oxygen radicals by stimulating the activation of the HPA axis [7]. Brain tissue is vulnerable to oxidative damage and increases the damage to the antioxidant defense system and apoptosis through various pathways such as DNA disruption and lipid peroxidation [83]. Chronic stress increases the release of inflammatory cytokines such as TNF-α and IL-1β, which increases cerebral oxidative stress, resulting in more cytokines [84]. Inflammatory cytokines and stress hormones activate MAPK, such as JNK and p38, through TNF-α receptor 1, and increase caspase-1 related to the production of inflammasome [15]. In particular, phosphorylated JNK promotes inflammatory response and apoptosis by reducing the ratio of Bcl-2 and Bax [85]. The increase in Bax/Bcl-2 ratio induced by chronic stress stimulates apoptotic signal cascade in mitochondria, and it upregulates the inflammatory protein expression levels of caspase-3, IL-1β, and TNF-α in the prefrontal cortex and hippocampus [41]. Thus, chronic stress causes damage to brain tissue through stimuli related to neuroinflammation and cytotoxicity, ultimately resulting in behavioral disorders [84]. In this study, chronic stress exposure increased the inflammatory response and cellular cytotoxicity in brain tissue; however, EFCS downregulated the inflammatory expression levels of caspase-1, TNF-α, IL-1β, p-JNK, Bax, and p-tau in brain tissues (Figure 8 and Figure 9). Similar to these studies, sprouts of *Cedrela sinensis* suppressed the levels of cytokines such as IL-6 and TNF-α and increased antioxidative enzymes such as glutathione peroxidase (GPx), glutathione S-transferase (GST), and SOD levels [86]. Rutin contained in *Cedrela sinensis* significantly decreased p-JNK, Bax, and caspase-3 levels in CdCl_2_-treated rat brain [87]. Rutin inhibited the TNF-α and IL-1β levels in fluoride-induced neurocytotoxicity in rat cerebrum and striatum [88]. Rutin regulated the inflammatory protein of p-NF-κB, TNF-α, and Nrf2, and apoptotic protein of caspase-3, Bax, and Bcl-2 in colistin-induced rat brain [89]. *Dendropanax morbifera*, which contains a large amount of rutin, downregulated the cerebral p-JNK and p-tau in high-fat-diet-induced diabetic mice [90]. In addition, isoquercitrin contained in the EFCD significantly reduced the level of protein expression of apoptosis markers, including Bax, caspase-3, and neuronal nitric oxide synthase (nNOS) against high-glucose-induced apoptosis in human cord venous endothelial cells (HUVEC) [91]. Quercitrin significantly reduced the production of TNF-α and IL-6 in the hippocampus in carbon tetrachloride (CCl_4_)-treated mice [92]. These results suggest that EFCS regulates stress-related factors and the expression of apoptosis factors in brain tissue. Therefore, EFCS containing various bioactive compounds can be used as a functional food material that can help with antidepression by regulating CUMS-induced inflammatory factors and cytotoxicity proteins in the brain.

## 5. Conclusions

In this study, ethyl acetate fraction from *Cedrela sinensis* (EFCS) in C57BL/6 mice was confirmed to have an ameliorating effect on excessive inflammatory and hormonal imbalance by CUMS. EFCS, which contains a large number of physiological compounds, protected neuronal cells against H_2_O_2_^−^ and corticosterone-induced cytotoxicity. EFCS improved anhedonia in the sucrose preference test (SPT), forced swimming test (FST), open field test (OFT), and tail suspension test (TST). EFCS inhibited mitochondrial dysfunction in brain tissue. EFCS ameliorated hypothalamic monoamine hormones by regulating the stress factors such as CRF, ACTH, and CYP11B1. Furthermore, EFCS modulated protein expression levels associated with inflammation and apoptosis factors in brain tissues. In conclusion, it is proposed that *Cedrela sinensis* could be used as a natural plant material to improve CUMS-induced depression-like behaviors by regulating inflammatory cytokines.

## Figures and Tables

**Table 1:** Chronic unpredictable mild stress (CUMS) model.

Stressors	Time	Description
Cage swap	24 h	Mice were exchanged from their cage to a stranger cage with other mice.
Cage tilting	24 h	Each cage was tilted by leaning at a 45° angle on the cage.
Empty cage	24 h	Mice were housed in empty cages without sawdust.
Food or water deprivation	24 h	Food or water was diverted.
Mild restraint	2 h	Mice were placed in the plastic box (10.5 cm width × 10.5 cm length × 5.5 cm height) with holes in every side except the bottom and top to breathe.
Overnight light exposure	24 h	Mice were exposed to light stress.
Wet bedding	24 h	Mice were housed in cages with added distilled water (200 mL).

**Table 2:** Identification of main compounds of ethyl acetate fraction from *Cedrela sinensis* (EFCS).

No.	RT ^(1)^ (min)	Parent Iron ^(2)^ (*m*/*z*)	MS^E^ Fragment ^(3)^ (*m*/*z*)	Compound
1	3.71	609.14	301.04, 151.00	Rutin
2	3.82	463.09	300.02, 301.03	Isoquercitrin
3	3.91	197.04	169.01, 124.01	Ethyl gallate
4	4.05	447.09	301.03, 271.02, 179.00	Quercitrin
5	4.28	431.10	285.04, 284.03	Kaempferol-3-O-rhamnoside
6	4.50	349.05	197.04, 169.01	Ethyl digallate

^(1)^ RT means retention time. ^(2)^ Ions are presented at m/z [M-H]^−^. ^(3)^ Bold indicates the main fragment of MS^E^.

## Data Availability

The data underlying this article are shared upon reasonable request to the corresponding author.

## Supplementary Materials

The following supporting information can be downloaded at: https://www.mdpi.com/article/10.3390/antiox11122448/s1, Table S1: Antioxidant capacity of ethyl acetate fraction from *Cedrela sinensis* (EFCS). Table S2: Information of primary antibody used in Western blotting. Figure S1: Cell viability of ethyl acetate fraction from *Cedrela sinensis* (EFCS).
