# Supplementary material for: Leaves of Cedrela sinensis Attenuate Chronic Unpredictable Mild Stress-Induced Depression-like Behavior via Regulation of Hormonal and Inflammatory Imbalance
Source: Antioxidants (Basel). 2022 Dec 12;11(12):2448. doi: 10.3390/antiox11122448 (PMC9774296; doi:10.3390/antiox11122448)
Supplement: Supplementary file 1 [file antioxidants-11-02448-s001.zip › antioxidants-2066552-supplementary.pdf]

Table S1. Antioxidant capacity of ethyl acetate fraction from *Cedrela sinensis* (EFCS).

TPC [a]	TFC [b]	ABTS [c]	MDA [d]	FRAP [e]
670.49 ± 3.70	337.09 ± 3.57	77.64 ± 1.12	0.97 ± 0.01	2.03 ± 0.01

[a] TPC, total phenolic content; [b] TFC, total flavonoid content; [c] ABTS, ABTS radical scavenging activity; [d] MDA, inhibitory effect of malondialdehyde (MDA); [e] FRAP, ferric ion reducing antioxidant power. Results are mean ± standard deviation (SD) (n = 3). Results of TPC and TFC are presented as mg of GAE/g and mg of RE/g, respectively. Results of ABTS radical scavenging activity, and inhibitory effect of MDA are presented as IC_{50} value (μg/mL). Result of FRAP are presented as absorbance value at 200 μg/mL.

Table S2. Information of primary antibody used in western blotting.

Antibody	Catalog # or code	Dilution used	Manufacture
β-actin	sc-69879	1:2,000	Santa Cruz Biotechnology, Inc. (Dallas, TX, USA)
CRF	sc-293187	1:1,000	Santa Cruz Biotechnology, Inc. (Dallas, TX, USA)
ACTH	sc-57018	1:1,000	Santa Cruz Biotechnology, Inc. (Dallas, TX, USA)
CYP11B1	sc-374096	1:1,000	Santa Cruz Biotechnology, Inc. (Dallas, TX, USA)
Capase-1	sc-392736	1:500	Santa Cruz Biotechnology, Inc. (Dallas, TX, USA)
TNF-α	sc-33639	1:1,000	Santa Cruz Biotechnology, Inc. (Dallas, TX, USA)
IL-1β	sc-515598	1:500	Santa Cruz Biotechnology, Inc. (Dallas, TX, USA)
Bax	sc-7480	1:2,000	Santa Cruz Biotechnology, Inc. (Dallas, TX, USA)
p-JNK	sc-6254	1:1,000	Santa Cruz Biotechnology, Inc. (Dallas, TX, USA)
p-tau	sc-12952	1:1,000	Santa Cruz Biotechnology, Inc. (Dallas, TX, USA)
BDNF	CSB-PA05775A0Rb	1:1,000	CusaBio Technology LLC (Houston, TX, USA)

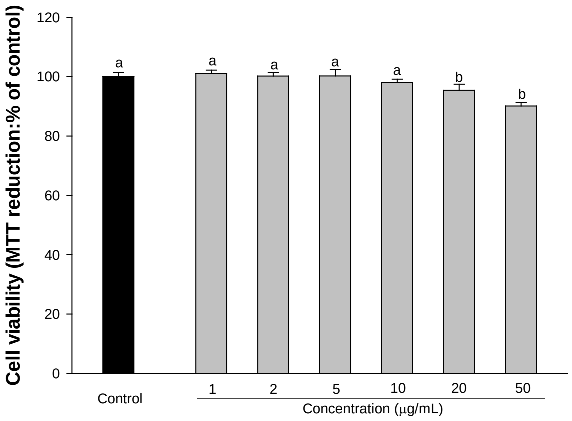

Figure S1. Cell viability of ethyl acetate fraction from *Cedrela sinensis* (EFCS). Results shown are mean ± SD (n=5). Data were statistically represented at $p<0.05$, and different lowercase letters indicate statistical significance.
